# Ascaris Lumbricoides Diagnosed During Evaluation of Iron Deficiency Anemia by Capsule Endoscopy

**DOI:** 10.7759/cureus.25208

**Published:** 2022-05-22

**Authors:** Polina Gaisinskaya, Samantha Sugerik, Christopher M Gebara

**Affiliations:** 1 Internal Medicine, Florida Atlantic University, Florida, USA

**Keywords:** anemia, ascaris lumbricoides, video capsule endoscopy, iron-deficiency anemia, capsule endoscopy

## Abstract

Iron deficiency anemia (IDA) is a common medical condition that has numerous possible causes, which include reduced dietary intake, blood loss, or reduced absorption, and often results in a referral to a gastroenterologist for further work-up. Reduced absorption of iron is an uncommon cause of iron deficiency in healthy non-vegetarian individuals and resource-rich countries. We present an uncommon cause of IDA in the western hemisphere, secondary to Ascaris lumbricoides infection in a 65-year-old female without a history of travel to endemic regions.

## Introduction

Iron deficiency anemia (IDA) is one of the most common causes of referral to a gastroenterologist. Common causes of IDA include decreased dietary intake, reduced absorption, and blood loss, though reduced absorption is often the least frequently implicated among these causes. Malabsorption is an especially rare cause of iron deficiency in resource-rich countries, and only a portion of those limited cases can be attributed to parasitic infections [1]. Ascaris infection is one such case in which infection has demonstrated influence on host nutritional status. In most cases, however, IDA is assumed to be caused by blood loss until proven otherwise, especially in middle-aged and older adults. In women, gynecologic causes are also common etiologies of IDA. Workup of iron deficiency anemia often involves consultation with gastroenterology as well as imaging for further investigation [1]. Of concern, of course, especially in older populations, is the possibility of an occult malignancy. Malabsorption in resource-rich areas raises concern for a physiologic disease impairing nutrient absorption or, in rare cases, an opportunistic infection feeding off the nutrients that the patient ingests. While Ascaris is not an uncommon infection, with about 819 million cases worldwide, it is rarely seen in resource-rich countries, and is more often seen in association with areas of low socioeconomic status and malnutrition and is especially unexpected in the absence of travel to endemic regions [2]. Many cases of Ascaris are asymptomatic, although symptoms such as abdominal bloating and constipation can also be seen, as the adult worms reside in the small intestine. Other possible manifestations of symptomatic infection include pulmonary symptoms and eosinophilic pneumonia secondary to the migration of the larvae to the lungs from the bloodstream and back into the gastrointestinal tract. Chronic infection is more often seen in conjunction with nutritional compromise in relation to the size of worm burden [1]. Additionally, children are more often severely affected by infection as compared to adults [3]. We present an unexpected case of iron deficiency anemia secondary to Ascaris lumbricoides infection, diagnosed by capsule endoscopy, in a 65-year-old female located in southern Florida, in the absence of travel to endemic regions for this helminth infection. This case was presented as an abstract poster at the American College of Gastroenterology Annual Scientific Meeting 2021 [4].

## Case presentation

**Figure 1:**
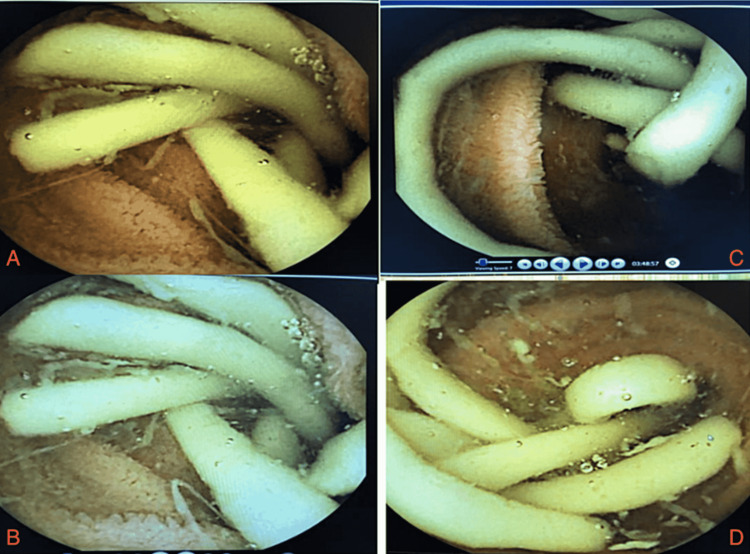
Panels A-D revealed the live viewer for the capsule endoscopy, which captured the large roundworm located in the terminal ileum

A 65-year-old postmenopausal female with a past medical history of hypothyroidism, non-insulin-dependent diabetes mellitus, and diverticulitis presented to the gastroenterology office for further evaluation of newly diagnosed iron-deficiency anemia found on routine laboratory work with subsequent iron studies for further delineation of the decreased hemoglobin. Further history revealed constipation with intermittent bloating for several days. She also reported straining to defecate secondary to constipation, which was associated with occasional visible red blood in her stool. She denied any long-term history of these symptoms. She denied black or tarry appearing stools and was otherwise asymptomatic on review of systems. She had no personal history of malignancy with appropriate screening or post-menopausal bleeding. She also denied any dietary restrictions that could contribute to decreased iron levels. A physical exam of the patient was negative for any pallor of abdominal findings. Colonoscopy was obtained for further workup based on the patient’s large intestinal complaints in the setting of the anemia and revealed only minor diverticulosis of the sigmoid colon without visible malignancy or other sources of bleeding. Subsequent esophagogastroduodenoscopy revealed esophageal erosion at the gastroesophageal junction and severe gastritis with no duodenitis. Because no source of bleeding that could explain the iron-deficiency anemia was identified, and because of the patient’s recent changes in bowel habits, capsule endoscopy was performed for further evaluation. Endoscopy imaging revealed a large worm in the distal ileum (Figure 1), believed to be Ascaris lumbricoides due to its size and appearance. The patient had been a long-term Florida resident for roughly 11 years since moving from New York City. Due to the pandemic, she had not traveled in over three years, denied exposure to any unsanitary conditions or free-standing water, and lived alone without any pets or sick contacts. She was treated with a one-time dose of 500 mg mebendazole with a resolution of the iron deficiency anemia and associated gastrointestinal symptoms on follow-up four weeks later. Repeat capsule endoscopy revealed no further worm burden throughout her gastrointestinal tract.

## Discussion

Ascaris lumbricoides is also known as the “human roundworm” and is one of the most common parasites in the world [1]. Humans are infected by fecal-oral transmission, specifically via ingestion of food or water contaminated by infective eggs [1]. Open defecation in developing countries is the major risk factor, and it is uncommon in developed countries due to modern sanitation. A small number of cases have also been linked to contact with domestic pigs, even in countries not typically associated with soil-transmitted helminth infections like Ascaris [2]. After ingestion, larvae hatch in the intestine, penetrate the intestinal mucosa to enter the bloodstream, and migrate to the pulmonary circulation. The larvae then cross the alveolar wall, travel to the larynx, and are swallowed again [2]. The worms mature and live within the lumen of the small intestine, obtaining nutrition from ingested food. About two to three months after the initial ingestion, female worms produce thousands of eggs daily that subsequently pass into the stool [2]. The eggs remain viable in warm, moist soil for years, with unfertilized eggs developing into infective embryonated eggs in this setting. The worms themselves live and produce eggs for several years in the small intestine [2]. Most infections by Ascaris lumbricoides are asymptomatic, but symptoms such as abdominal pain, bloating, and intestinal obstruction are possible. Malnutrition can be seen in patients with a heavy worm burden, as the organism shares nutrition with the host [1]. This is associated with, but not limited to, iron deficiency, as seen in our case. Diagnosis typically requires direct visualization. In our case, this was achieved through capsule endoscopy after esophagogastroduodenoscopy, and colonoscopy did not reveal any source of the iron deficiency. This progression through imaging modality has been described in other cases of Ascaris, which were diagnosed by capsule endoscopy when other modalities failed to reveal any source of bleeding or obvious pathology [3,5]. Treatment is with albendazole 400 mg once or with mebendazole 500 mg once or 100 mg twice daily for three days [6,7].

## Conclusions

In patients with IDA and non-specific abdominal symptoms, evaluation for Ascariasis along with other intestinal helminth infections should be considered in the setting of applicable exposure or if all other common causes are ruled out. In some cases, parasitic infections may result in overt blood loss, which may prompt earlier endoscopic evaluation. As seen in our case, capsule endoscopy can be used to visualize the intestinal mucosa and investigate further. 
